# Effect of Corrective Exercise on Static Balance, Food Consumption, and Body Composition in the Early Period After Bariatric Surgery

**DOI:** 10.1007/s11695-024-07136-1

**Published:** 2024-03-06

**Authors:** Dilara Cetin, Meral Kucuk Yetgin, Ahmet Gokhan Turkcapar, Burke Koksalan, Sena Durmaz

**Affiliations:** 1 Department of Physical Education and Sports, Institute of Health Science, Marmara University, Istanbul, Türkiye; 2 Department of Coaching Education, Sport Health Sciences, Faculty of Sport Sciences, Marmara Üniversitesi Anadoluhisarı Yerleşkesi, Göksu Mah. Cuma Yolu Cad. No:1, Spor Bilimleri Fakültesi PK, Beykoz, Istanbul, 34815 Türkiye; 3 Turkcapar Bariatrics Obesity Center, Istanbul, Türkiye; 4 Department of Physical Therapy and Rehabilitation, Acıbadem Fulya Hospital, Istanbul, Türkiye

**Keywords:** Sleeve gastrectomy, Corrective exercise, Static balance, Body composition, Dietary intake

## Abstract

**Purpose:**

To determine the impact of corrective exercise program applied during the period of rapid weight loss following bariatric surgery on static balance, dietary intake, and body composition.

**Materials and Methods:**

Participants who had undergone Sleeve Gastrectomy (SG) surgery were divided into as Corrective Exercise Group (CEG), and Control Group (CG). CEG underwent a 12-week supervised corrective exercise program. Body composition and static balance of all participants were assessed before and after the study. Their physical activity levels and dietary intake were also evaluated.

**Results:**

Following of the corrective exercise program, both groups exhibited significant reductions in body weight, BMI, fat mass, fat percentage, muscle mass, waist circumference-to-height ratio, and visceral adiposity values (*p* < 0.05). Additionally, the CEG showed increase in lean body mass percentage (*p* < 0.001). In measurements related to static balance, values for eyes-closed perimeter (*p* = 0.015), eyes-closed (*p* = 0.006), eyes-open (*p* = 0.028) ellipses area, average F-B speed, and eyes-open center of pressure in the X-axis (C.O.P.X.) sway distance significantly decreased in both groups (*p* = 0.025). However, the difference in eyes-open C.O.P.X. sway distance was found to be higher in the CG (mean difference = 8.67;* p* = 0.034). Postoperatively, there were significant decreases in energy, protein, fat, CHO (carbohydrate), CHO percentage, fiber, and iron values, while protein percentage (*p* < 0.001), vitamin D (*p* = 0.003), and B12 (*p* < 0.001) values increased.

**Conclusion:**

It has been observed that the corrective exercise program implemented in the early postoperative period following SG surgery had a positive impact on eyes-open static balance.

**Graphical Abstract:**

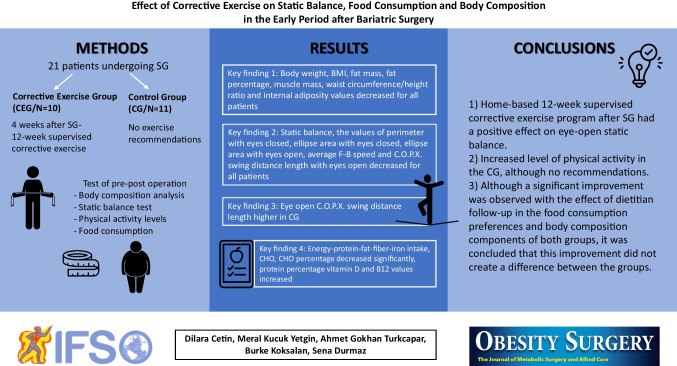

## Introduction

In the post-bariatric surgical period, obtaining nutritional support is essential to facilitate the participants’ adaptation to the new stomach-intestinal physiology and to preempt potential specific nutritional challenges [1]. Furthermore, numerous studies have indicated that exercise performed after bariatric surgery reduces the associated medical problems enhances levels of physical activity, improves quality of life, and reduces the risk of mortality [2, 3].

In individuals with obesity, the excessive fat mass in the abdominal region results in a forward shift of the center of gravity. Sarcopenia, which signifies progressive muscle loss associated with obesity, further disrupts static balance by altering the body’s center of gravity [4]. Hence, individuals with obesity may involuntarily deviate from their perceived point of balance. The ability to maintain static balance while standing is paramount for the effective and efficient execution of numerous daily activities [5]. It has been concluded that in women with overweight or obesity [6–8], there is an increased sway velocity in both eyes-open and eyes-closed conditions, indicating the adverse impact of obesity on postural stability. To the best of our knowledge, there is a limited body of research investigating static balance both in the preoperative period of bariatric surgery [9] and during the period of rapid weight loss following bariatric surgery [10]. However, there is currently no study available that examines the impact of corrective exercises on static balance in bariatric surgery participants after the surgery.

Corrective exercises are founded upon an anatomical, kinesiological, and biomechanical model, aiming to enhance mobility and stability in daily life [11]. The implementation of these exercises has been reported to promote stability and postural alignment within the body by appropriately activating muscle groups through neuromuscular control [12].

Considering the potential for static balance disturbances due to the shift in the center of gravity, particularly among individuals with obesity who have undergone bariatric surgery [13], there arises a necessity for combined exercise programs, which encompass corrective and static balance exercises, within this population.

The aim of this study is to investigate the effects of a gradual increase in intensity over a 12-week period (initiated in the first month post-surgery, 3 days per week; 40–70 min per day, totaling 120–210 min per week) of corrective exercise on body composition, dietary preferences, and static balance during the early post-bariatric surgery phase.

## Method

### Study Participants

The study enrolled 21 adult female participants aged 20–60, with a BMI ≥ 30 kg/m^2^, who underwent SG surgery in a private clinic between May 2022 and December 2022.

The participants with similar age and BMI characteristics and who were not currently engaged in any other exercise program were recruited. Those participants who were willing to join a regular exercise program assigned to Corrective Exercise Group (CEG), while those who were not willing to join a regular exercise program were included in Control Group (CG).

Initially each study group included 11 participants, one participant in CEG was lost to follow-up after the surgery. The study was completed with a total of 21 participants as 10 participants in CEG (BMI = 42.13 kg/m^2^) and 11 participants in the CG (BMI = 42.17 kg/m^2^).

Study inclusion criteria were undergoing SG surgery, not engaging in any other exercise program for CG, being able to participate in an exercise program for 3 days a week and actively participating in online communication through the WhatsApp platform for CEG. Those participants who participated in final test measurements, which took place in a single center, included in this study.

The exclusion criteria were having any disabling disease at the time of measurements, having a physical disability that would hinder participation in a corrective exercise program, having a permanent disability, having diseases of the lower extremities, having undergone orthopedic surgery or currently undergoing treatment in this regard, and being male.

Ethical approval for the study was obtained from the Marmara University Clinical Research Ethics Committee (Protocol No: 2022/13–01).

### Data Collection Methods

In the week preceding the SG surgery, all participants underwent data collection, including demographic information and measurements of body composition (body fat percentage, body fat mass, body muscle mass, visceral fat score), as well as anthropometric measurements (BMI, body weight, height, waist-to-height ratio).

Static balance measurements were conducted under the supervision of a physiotherapist, both with eyes-open and eyes-closed. Additionally, the International Physical Activity Questionnaire (IPAQ), Food Frequency Form, and Food Intake Record Form were administered. At the 4th month post SG surgery, all participants repeated all questionnaires and measures except the demographic information form, using the same methods as before surgery.

### Anthropometric Measurements and Assessment of Body Composition

Height measurements were taken barefoot using a stadiometer (SECA, Germany) with a precision of 0.01 mm, while body weights were measured using a body composition analyzer, TANITA MC-580, with a precision of 0.1 kg. Body composition measurements, including body mass index (BMI), body fat mass (kg), body fat percentage (%), lean body mass (kg), lean body percentage (%), waist-to-height ratio, and visceral fat score, were analyzed using a Bioelectrical impedance analysis (BIA) device, and the waist-to-height ratio measurement reported by the Tanita MC-580 was accepted as within the healthy range between 0.4 and 0.49. However, the range of 0.5 to 0.59 is considered indicative of health risks associated with high body weight, while 0.6 or higher is assessed as a higher risk of health issues [14]. Tanita displays the visceral fat percentage scale from 1 to 59. A range of 1–12 indicates healthy levels of visceral fats, 13 and above is indicative of excess visceral fat and potential health risks. During the classification of obesity, the criteria for BMI value were considered in accordance with the World Health Organization’s classification. According to these criteria, BMI values of 30 kg/m^2^ and above were classified as first-degree obesity, 35 kg/m^2^ and above as second-degree obesity, and 40 kg/m^2^ and above as third-degree obesity [15].

### Static Balance Measurement

The objective of the static balance test is to measure changes in the position of the center of gravity over time. For this purpose, static balance performance was assessed using the isokinetic balance measurement platform (PROKIN 252, Tecnobody, Bergamo, Italy). The tests were conducted with participants standing barefoot, both with eyes-open and eyes-closed. The position of the feet was determined to be equidistant from the origin point, using the lines on the platform’s X and Y axes as references. Participants were instructed to focus their gaze on a fixed point marked at a distance of 1 m in front of them. The arms were placed adjacent to the body, while a brief resting period of approximately 40 s was allowed between test measurements, each spanning 30 s. Each measurement was repeated twice, and the average values of the results were calculated. The assessment of static balance was performed based on data including the average center of pressure in the x-axis (average C.O.P.X) in millimeters, the average center of pressure in the y-axis (average C.O.P.Y) in millimeters, lateral sway speed (medium-lateral speed) in millimeters per second, forward–backward sway speed (average forward–backward speed) in millimeters per second, perimeter used (perimeter-P) in millimeters, and the area used (ellipse area) in square millimeters. This data was utilized to derive the static balance score for each participant. Static balance measurements were taken by an expert physiotherapist who is a researcher at the Physical Therapy and Rehabilitation Center of a hospital.

### International Physical Activity Questionnaire (IPAQ)

The IPAQ is a subjective instrument used to measure the physical activity levels of individuals who participated in the survey, developed by Craig in 2003 [16]. The Turkish validity and reliability of the questionnaire were carried out by Ozturk in 2005 [17]. IPAQ is used in two ways, short and long. In this research, a short form consisting of 7 questions was used. The 7 questions in the questionnaire were prepared to determine the duration of walking, moderate and high-intensity activities, and sitting times of individuals in daily life, based on days and hours. The answers given to the questionnaire determine the metabolic equivalent (ME-min) amounts of individuals in minutes as physical activity duration (minutes) and frequency (days). After the MET (metabolic equivalent) values of individuals, vigorous physical activity is calculated as 8.0 MET, moderate physical activity: 4.0 MET, low physical activity: 3.3 MET, and walking: 1.5 MET. The total MET-minutes/week = (walking + moderate intensity + vigorous intensity + sitting) MET-minutes/week is calculated with this formula. According to the MET scores, the physical activity level of the individuals is divided into groups as low (< 600 MET min/week), moderate (600–3000 MET min/week), and vigorous (> 3000 MET min/week) [17].

### Food Frequency Questionnaire

Participants were queried about the frequency of consumption of each of the 39 listed foods in the food frequency questionnaire, both before the SG surgery and at the 4th month post-surgery. Food consumption frequency was categorized into seven options: daily, 5–6 times per week, 3–4 times per week, 1–2 times per week, once every 15 days, once a month, and never. Foods in this questionnaire were grouped under the titles as “milk and dairy products, meat, legumes, bread, fat, beverages, sugary products, others.” Foods were detailed under each group, e.g., whole, semi skimmed, or skimmed for milk.

### Food Record

To determine the participants’ nutritional status, a 24-h dietary recall was obtained, and the analyses were conducted using the BeBiS (Beslenme Bilgi Sistemleri) 8.2 program. Examination of participants’ energy, carbohydrate, protein, fat, fiber, and fluid intake was carried out based on group-appropriate averages according to the recommendations outlined in the Türkiye Dietary Guide (TÜBER) of 2015 [18].

### Diet Programs

After the SG surgery, all participants received guidance from a bariatric dietitian and were monitored online for a period of 4 months. Both groups were advised to consume two sachets of 100% isolated whey protein specially formulated for bariatric surgery participants daily for the first 4 months following the SG surgery. Protein requirements during the initial 4 months were calculated as 60–80 g/day or 1–1.5 g/kg (ideal body weight), with the recommendation of isolated whey protein powder support when necessary [19]. From the second day after SG surgery, the participants started to take a protein powder supplement suitable for consumption after bariatric surgery, which is a 100% isolated whey protein supplement suitable for bariatric surgery. This protein supplement consists of 100% isolated whey protein, probiotic fiber and a blend of 21 vitamins and minerals.

### Corrective Exercise Program

Both study groups were advised to maintain physical activity (such as walking) in the first month following the surgery. The CEG group underwent a 12-week corrective exercise program starting from the 4th week after surgery. Regular exercise programs, in the form of training video material prepared by a certified exercise specialist, were provided to the CEG participants. The exercises were conducted three days a week. The adherence to the program was monitored with the photos of each session shared by the participants. A WhatsApp group was created for the CEG participants share their photos while exercising. In the event of missing the exercise session, participants were provided with makeup sessions during the rest of the week.

The design of the exercise program for the study drew upon corrective exercise resources found in the literature [20, 21]. The corrective exercise program was structured to span a total of 12 weeks, comprising 36 sessions in total (3 days/week, approximately 18.6–50 min per day). Over the course of the 12 weeks, the training program was organized into three phases: warm-up (8–10 min), main phase (26–53 min), and cool-down phase (5 min). The program was divided into two phases, each lasting 6 weeks. In Phase 1, the total duration of a single training session was approximately 39 min in weeks 1–2, ~ 43 min in weeks 3–4, and ~ 48 min in weeks 5–6. Phase 2, on the other hand, had a total duration of ~ 50.5 min in weeks 7–8, 58 min in weeks 9–10, and a total of 68 min for weeks 11 and 12. The warm-up phase was 8 min in Phase 1 and 10 min in Phase 2, while the cool-down phase lasted for 5 min. Both the warm-up and cool-down segments were focused on the targeted muscle groups for the exercises. In this program, corrective exercises utilized body weight as well as resistance bands, free weights, and textured foam rollers. The intensity of exercises involving resistance bands was determined based on the elasticity level of the bands (Thera-band; Hygenic Co., Akron, OH, USA). In Phase 1, red and green bands were used, while in Phase 2, green and blue bands were employed. To ensure the safe engagement of patients with obesity in exercises, the kilograms of free weights were determined using the one-repetition maximum (1RM) method, and the weights used over the course of three months ranged from 1 to 5 kg. Exercises were performed using body weight, resistance bands, free weights, and textured foam rollers (Table 1).

**Table 1:** Corrective exercise program

Phases	Phase 1			Phase 2		
Weeks	1–2	3–4	5–6	7–8	9–10	11–12
Movement 1	^a^Wall thoracic rotation ^b^Overhead stretch on foam roller			^a^Prone overhead press ^b^Prone T raise		
Movement 2	^a^Resisted bird dog ^b^Russian twist			^a^Anti-rotation walk ups ^b^Resisted lateral shifting		
Movement 3	^a^Ball crunch ^b^Sit-up			^a^Flutter kick ^b^Scissor flutter kick		
Movement 4	^a^Resisted plantarflexion ^b^Resisted dorsiflexion			^a^Band Pulses Overhead ^b^Bilateral External Rotation with Band		
Movement 5	^a^Adductor rock back ^b^Frog pump with band			^a^Banded Hip Thrust ^b^90/90 Hip Thrust		
Movement 6	^a^Biceps curl overhead press ^b^Overhead triceps extension			Biceps curl overhead press + Overhead extension		
Movement 7	Dowel hip hinge			Mountain climber		
Movement 8	Spanish squat			Step up with knee		
Movement 9				^a^Dumbbell lying chest fly ^b^Dumbbell bent over row		
Movement 10				Heel raise with ball squeeze		

^a^Exercise to be done in the first 2 weeks of each phase. ^b^Exercise to be done in the last 4 weeks of each phase

Control group was established from participants who indicated at the beginning of the study thay they did not want to participate in any exercise intervention after surgery. They were not subjected to any exercise program during the 12 weeks of study. They were monitored by asking them if they did participate in any exercise program during their appointments with the nutritionist.

### Statistical Analysis

Descriptive statistics including mean, standard deviation (SD), median, interquartile range (IQR: 25th–75th percentiles), frequency, and percentage were presented for statistical analysis. The assumption of normal distribution was assessed using the Shapiro–Wilk test. Wilcoxon test was applied for within-group comparisons. The comparison of study groups before surgery was assessed using independent samples *T*-test, Mann–Whitney *U* test, and Fisher’s exact test. The post-surgery physical activity level was compared between CEG and CG with and Pearson chi-square test. Changes in measurements before surgery and after corrective exercise were examined using two-way (group × time) analysis of variance (ANOVA) in repeated measures, and for variables where parametric assumptions were not met, non-parametric analysis was conducted using aligned rank transformation. The Bonferroni *p*-value, mean difference, and standard error (SE) were reported for the pairwise comparisons when the group and time interaction was statistically significant in two-way ANOVA. Statistical significance was accepted at *p* < 0.05. Effect sizes were presented using Cohen’s *d* (small: 0.2–0.5; medium: 0.5–0.8; large: ≥ 0.8), rank-biserial correlation coefficient (${r}_{{\text{rb}}}$ value indicating no difference between groups: 0; first group higher than the second group: > 0; second group lower than the first group: < 0), and partial eta-squared (*η*^2^_p_ value indicating low: 0.01–0.06; medium: 0.06–0.14; high: ≥ 0.14) [22, 23]. Statistical analyses were performed using R software version 4.2.1 with the ARTools package [24, 25].

The power analysis was based on the parameters of the study by Picó-Sirvent et al., 2019 [26], which examined the effects of different exercise methods in individuals with obesity. The power analysis aimed to achieve 95% power, at a significance level of α = 0.05 and the effect size of *d* = 1.62, resulted in minimum required sample size of 11 participants per group using the G*Power (v3.1.9) software. Initially it was planned for a minimum of 11 participants in each group, the groups were completed with a total of 21 participants, consisting of CEG (*n* = 10) and CG (*n* = 11), due to one participant not being able to complete the tests.

## Results

### Participant Baseline Characteristics

The mean age of the participants was 40.80 (SD = 10.52) years in CEG group and 37.64 (SD = 11.00) years in CG group (*p* > 0.05). The study groups had similar education level, smoking habits, cholesterol level, LDL level, fasting blood glucose levels, IPAQ scores, and physical activity level (*p* > 0.05). Those participants in CEG group had higher HDL level than CG group (*p* < 0.05). The presence of menopause, MI story, hypertension, and drug use were similar between the study groups (*p* > 0.05) (Appendix Table 7).

### Changes in Body Composition and Anthropometric Measurements

Four months post-surgery, participants showed a significant decrease in body weight, BMI, fat mass, fat percentage, and visceral fat score, with a concurrent significant increase in lean mass percentage compared to pre-surgery measurements (*p* < 0.05) (Table 2).

**Table 2:** Post surgery changes in body composition and anthropometric measurements

Variable	Group	Pre-op		Post-op 4th month		Effect (*F*;* p*; ${{\varvec{\eta}}}_{\mathbf{p}}^{2}$)^a^		
		Mean ± SD	Median (IQR)	Mean ± SD	Median (IQR)	Group	Time	*G* × *T*
Body mass (kg)	CEG	111.54 ± 13.34	112 (103–115)	83.07 ± 10.23	80.45 (76.8–87.1)	*F* = 0.077; *p* = 0.758; ${\eta }_{{\text{p}}}^{2}$ = 0.004	***F*** = **611.204; *p* < 0.001;** ${{\varvec{\eta}}}_{\mathbf{p}}^{2}$ = **0.970**	*F* = 2.176; *p* = 0.157; ${\eta }_{{\text{p}}}^{2}$ = 0.103
	CG	111.25 ± 11.95	115 (102–119.1)	85.98 ± 8.39	89.2 (78.6–91)			
BMI (kg/m^2^)	CEG	42.13 ± 3.42	42.48 (38.28–43.51)	31.4 ± 2.91	30.47 (29.77–32.38)	*F* = 0.109; *p* = 0.745; ${\eta }_{{\text{p}}}^{2}$ = 0.006	***F*** = **638.443; *p* < 0.001;** ${{\varvec{\eta}}}_{\mathbf{p}}^{2}$ = **0.971**	*F* = 2.051; *p* = 0.168; ${\eta }_{{\text{p}}}^{2}$ = 0.097
	CG	42.17 ± 5.88	40.86 (37.49–48.77)	32.59 ± 4.27	32.21 (30.32–35.77)			
Fat mass (kg)	CEG	51.89 ± 9.69	50.75 (45.4–56.1)	29.9 ± 8.77	28.35 (23.9–34)	*F* = 0.031; *p* = 0.862; ${\eta }_{{\text{p}}}^{2}$ = 0.002	***F*** = **512.096; *p* < 0.001;** ${{\varvec{\eta}}}_{\mathbf{p}}^{2}$ = **0.964**	*F* = 1.064; *p* = 0.315; ${\eta }_{{\text{p}}}^{2}$ = 0.053
	CG	51.61 ± 9.62	53.1 (42.4–59.2)	31.54 ± 8.01	33.2 (26.5–33.7)			
Fat percentage (%)	CEG	46.4 ± 3.01	45.85 (44.48–46.82)	35.31 ± 5.82	35.47 (31.56–39.04)	*F* = 0.007; *p* = 0.934; ${\eta }_{{\text{p}}}^{2}$ = 0.001	***F*** = **4.790;** *p* = **0.041;** ${{\varvec{\eta}}}_{\mathbf{p}}^{2}$ = **0.201**	*F* = 0.473; *p* = 0.500; ${\eta }_{{\text{p}}}^{2}$ = 0.024
	CG	46.39 ± 3.88	46.25 (44.1–50.04)	37.2 ± 5.68	37.33 (35.71–38.9)			
Muscle mass^b^ (kg)	CEG	56.25 ± 4.16	56.8 (54.2–58.6)	50.42 ± 2.63	51.3 (48.2–52.7)	*F* = 0.012; *p* = 0.914; ${\eta }_{{\text{p}}}^{2}$ = 0.001	***F*** = **94.027; *p* < 0.001;** ${{\varvec{\eta}}}_{\mathbf{p}}^{2}$ = **0.830**	*F* = 1.358; *p* = 0.258; ${\eta }_{{\text{p}}}^{2}$ = 0.070
	CG	55.85 ± 3.66	55.5 (52.1–58.6)	51.13 ± 2.7	52.1 (49.4–54)			
Muscle percentage (%)^b^	CEG	50.9 ± 2.85	51.43 (50.49–52.67)	61.51 ± 5.56	61.43 (57.94–64.99)	*F* = 1.404; *p* = 0.251; ${\eta }_{{\text{p}}}^{2}$ = 0.070	***F*** = **61.787; *p* < 0.001;** ${{\varvec{\eta}}}_{\mathbf{p}}^{2}$ = **0.760**	*F* = 4.104; *p* = 0.057; ${\eta }_{{\text{p}}}^{2}$ = 0.018
	CG	50.89 ± 3.67	51.05 (47.46–53.12)	56.78 ± 10.08	58.58 (51.93–61.02)			
Waist circumference/height ratio	CEG	0.79 ± 0.07	0.8 (0.73–0.82)	0.59 ± 0.07	0.6 (0.53–0.62)	*F* = 0.156; *p* = 0.697; ${\eta }_{{\text{p}}}^{2}$ = 0.008	***F*** = **258.994; *p* < 0.001;** ${{\varvec{\eta}}}_{\mathbf{p}}^{2}$ = **0.932**	*F* = 1.494; *p* = 0.237; ${\eta }_{{\text{p}}}^{2}$ = 0.073
	CG	0.79 ± 0.1	0.78 (0.69–0.91)	0.62 ± 0.09	0.62 (0.6–0.7)			
Visceral adiposity score^b^	CEG	13.3 ± 2.11	13.5 (12–15)	7.3 ± 1.83	7.5 (6–9)	*F* = 0.055; *p* = 0.817; ${\eta }_{{\text{p}}}^{2}$ = 0.001	***F*** = **387.347; *p* < 0.001;** ${{\varvec{\eta}}}_{\mathbf{p}}^{2}$ = **0.950**	*F* = 1.617; *p* = 0.219; ${\eta }_{{\text{p}}}^{2}$ = 0.080
	CG	12.91 ± 2.66	14 (10–15)	7.45 ± 2.42	7 (6–9)			

^a^Two-way ANOVA in repeated measures ^b^with aligned rank transformation. *G*, group; *T*, time; *CEG*, corrective exercise group; *CG*, control group; *SD*, standard deviation; *IQR*, interquartile range

### Changes in Static Balance with Eyes-Open and Eyes-Closed Measurements

The static balance improved for the eyes-open measurements with the decrease in of C.O.P.X., the ellipse area and the average forward–backward speed (*p* < 0.05) (Table 3). The sway difference in C.O.P.X. was significantly lower in the CEG group than the CG group 4 months post-surgery (mean difference = 8.67; SE = 3.79; *t* = 2.291; *p* = 0.034; *d* = 1.00). The static balance improved for the eyes-closed measurements with the decrease in the ellipse area, the perimeter and the average forward–backward speed (*p* < 0.05) (Table 4). It was concluded that there was an improvement in static balance measurements in both groups.

**Table 3:** Post surgery changes in static balance with eyes-open measurements

Parameter	*G*	Pre-op		Post-op 4th month		Effect (*F*; *p*; ${{\varvec{\eta}}}_{\mathbf{p}}^{2}$)^a^		
		Mean ± SD	Median (IQR)	Mean ± SD	Median (IQR)	Group	Time	*G* × *T*
Eyes-open C.O.P.X.^a^ (mm)	CEG	2.60 ± 9.20	5.00 (− 4.25 to 8.50)	0.20 ± 6.30	− 0.50 (− 4.25 to 3.50)	*F* = 0.051; *p* = 0.824; ${\eta }_{{\text{p}}}^{2}$ = 0.002	*F* = 0.295; *p* = 0.593; ${\eta }_{{\text{p}}}^{2}$ = 0.020	***F*** = **5.878; *p*** = **0.025;** ${{\varvec{\eta}}}_{\mathbf{p}}^{2}$ = **0.240**
	CG	− 1.09 ± 6.80	− 1.00 (− 5.00 to 2.00)	5.18 ± 4.81	4.00 (2.00–10.00)			
Eyes-open C.O.P.Y.^a^ (mm)	CEG	8.00 ± 26.47	3.00 (− 18.25 to 28.25)	− 4.40 ± 24.39	− 4.50 (− 23.25 to 17.00)	*F* = 0.574; *p* = 0.458; ${\eta }_{{\text{p}}}^{2}$ = 0.030	*F* = 1.677; *p* = 0.211; ${\eta }_{{\text{p}}}^{2}$ = 0.080	*F* = 0.031; *p* = 0.862; ${\eta }_{{\text{p}}}^{2}$ = 0.001
	CG	− 0.46 ± 34.59	− 2.00 (− 24.00 to 31.00)	− 11.64 ± 26.94	− 4.00 (− 32.00 to 12.00)			
Eyes-open ellipse area^a^ (mm^2^)	CEG	188.70 ± 96.01	178.50 (93.75–263.75)	145.90 ± 79.80	110.00 (80.50–219.75)	*F* = 0.340; *p* = 0.567; ${\eta }_{{\text{p}}}^{2}$ = 0.020	***F*** = **5.654; *p*** = **0.028;** ${{\varvec{\eta}}}_{\mathbf{p}}^{2}$ = **0.230**	*F* = 0.037; *p* = 0.850; ${\eta }_{{\text{p}}}^{2}$ = 0.001
	CG	221.18 ± 136.76	196.00 (155.00–232.00)	160.27 ± 79.37	162.00 (102.00–215.00)			
Eyes-open perimeter^a^ (mm)	CEG	288.50 ± 43.24	295.50 (260.00–316.25)	266.50 ± 35.17	266.00 (232.75–288.25)	*F* = 0.522; *p* = 0.479; ${\eta }_{{\text{p}}}^{2}$ = 0.030	*F* = 0.680; *p* = 0.420; ${\eta }_{{\text{p}}}^{2}$ = 0.030	*F* = 0.920; *p* = 0.349; ${\eta }_{{\text{p}}}^{2}$ = 0.050
	CG	294.46 ± 56.41	277.00 (258.00–330.00)	292.55 ± 38.87	280.00 (262.00–330.00)			
Eyes-open average F-B speed (mm/sn)	CEG	7.00 ± 1.05	7.00 (6.25–8.00)	6.10 ± 0.57	6.00 (6.00–6.00)	*F* = 0.641; *p* = 0.433; ${\eta }_{{\text{p}}}^{2}$ = 0.033	***F*** = **6.314; *p*** = **0.021;** ${{\varvec{\eta}}}_{\mathbf{p}}^{2}$ = **0.249**	*F* = 0.014; *p* = 0.906; ${\eta }_{{\text{p}}}^{2}$ = 0.001
	CG	7.27 ± 1.49	7.00 (6.00–8.00)	6.46 ± 1.37	6.00 (6.00–7.00)			
Eyes-open average M-L speed (mm/sn)	CEG	4.80 ± 1.14	5.00 (4.00–5.00)	4.60 ± 1.58	5.00 (4.00–5.00)	*F* = 0.256; *p* = 0.619; ${\eta }_{{\text{p}}}^{2}$ = 0.013	*F* = 0.077; *p* = 0.784; ${\eta }_{{\text{p}}}^{2}$ = 0.004	*F* = 0.077; *p* = 0.784; ${\eta }_{{\text{p}}}^{2}$ = 0.004
	CG	4.91 ± 1.22	5.00 (4.00–6.00)	4.91 ± 1.04	5.00 (4.00–6.00)			

^a^Two-way ANOVA in repeated measures with aligned rank transformation. *G*, group; *T*, time; *CEG*, corrective exercise group; *CG*, control group; *SD*, standard deviation; *IQR*, interquartile range

**Table 4:** Post surgery changes in static balance with eyes-closed measurements

Parameter	*G*	Pre-op		Post-op 4th month		Effect (*F*; *p*; ${{\varvec{\eta}}}_{\mathbf{p}}^{2}$)^a^		
		Mean ± SD	Median (IQR)	Mean ± SD	Median (IQR)	Group	Time	*G* × *T*
Eyes-closed C.O.P.X.^a^ (mm)	CEG	2.90 ± 8.81	3.50 (− 3.25 to 8.25)	1.30 ± 4.03	1.50 (− 2.25 to 5.25)	*F* = 0.468; *p* = 0.502; ${\eta }_{{\text{p}}}^{2}$ = 0.020	*F* = 0.142; *p* = 0.711; ${\eta }_{{\text{p}}}^{2}$ = 0.007	*F* = 1.469; *p* = 0.502; ${\eta }_{{\text{p}}}^{2}$ = 0.070
	CG	0.46 ± 5.80	− 1.00 (− 3.00 to 3.00)	1.91 ± 4.18	2.00 (− 2.00 to 6.00)			
Eyes-closed C.O.P.Y.^a^ (mm)	CEG	13.00 ± 29.66	11.50 (− 20.00 to 35.25)	0.20 ± 26.55	− 6.00 (− 18.75 to 28.00)	*F* = 1.059; *p* = 0.316; ${\eta }_{{\text{p}}}^{2}$ = 0.050	*F* = 0.231; *p* = 0.636; ${\eta }_{{\text{p}}}^{2}$ = 0.010	*F* = 0.226; *p* = 0.640; ${\eta }_{{\text{p}}}^{2}$ = 0.010
	CG	− 4.73 ± 30.74	− 1.00 (− 29.00 to 28.00)	− 4.46 ± 31.89	− 2.00 (− 38.00 to 25.00)			
Eyes-closed ellipse area^a^ (mm^2^)	CEG	589.90 ± 372.79	513.50 (305.75–746.50)	392.90 ± 228.66	350.00 (205.75–534.25)	*F* = 0.501; *p* = 0.488; ${\eta }_{{\text{p}}}^{2}$ = 0.030	***F*** = **9.650; *p*** = **0.006;** ${{\varvec{\eta}}}_{\mathbf{p}}^{2}$ = **0.340**	*F* = 0.001; *p* = 0.982; ${\eta }_{{\text{p}}}^{2}$ = 0.001
	CG	625.00 ± 243.14	560.00 (484.00–733.00)	467.00 ± 227.20	452.00 (269.00–572.00)			
Eyes-closed perimeter^a^ (mm)	CEG	521.40 ± 150.44	465.00 (425.50–558.25)	443.40 ± 103.14	431.00 (373.75–493.50)	*F* = 0.737; *p* = 0.401; ${\eta }_{{\text{p}}}^{2}$ = 0.040	***F*** = **7.170; *p*** = **0.015;** ${{\varvec{\eta}}}_{\mathbf{p}}^{2}$ = **0.270**	*F* = 0.004; *p* = 0.950; ${\eta }_{{\text{p}}}^{2}$ = 0.001
	CG	521.18 ± 70.98	538.00 (465.00–578.00)	478.82 ± 119.74	464.00 (400.00–519.00)			
Eyes-closed average F-B speed (mm/sn)	CEG	13.60 ± 4.27	11.50 (11.00–14.75)	10.50 ± 2.80	10.00 (9.00–11.00)	*F* = 0.256; *p* = 0.619; ${\eta }_{{\text{p}}}^{2}$ = 0.013	***F*** = **25.162; *p* < 0.001;** ${{\varvec{\eta}}}_{\mathbf{p}}^{2}$ = **0.570**	*F* = 0.057; *p* = 0.814; ${\eta }_{{\text{p}}}^{2}$ = 0.003
	CG	14.09 ± 1.92	14.00 (13.00–15.00)	11.27 ± 3.29	11.00 (10.00–12.50)			
Eyes-closed average M-L speed (mm/sn)	CEG	8.30 ± 3.27	8.00 (6.25–9.00)	7.70 ± 1.89	7.50 (7.00–8.00)	*F* = 0.208; *p* = 0.653; ${\eta }_{{\text{p}}}^{2}$ = 0.011	*F* = 0.412; *p* = 0.529; ${\eta }_{{\text{p}}}^{2}$ = 0.021	*F* = 0.224; *p* = 0.642; ${\eta }_{{\text{p}}}^{2}$ = 0.012
	CG	7.64 ± 1.91	8.00 (6.00–9.00)	7.55 ± 2.30	7.00 (5.50–9.00)			

^a^Two-way ANOVA in repeated measures with aligned rank transformation. *G*, group; *T*, time; *CEG*, corrective exercise group; *CG*, control group; *SD*, standard deviation; *IQR*, interquartile range

### Changes in Physical Activity Level

Post-surgery IPAQ score was significantly increased in both study groups (*p* < 0.001). The post-surgery physical activity level was significantly different between the exercise groups (chi-square = 8.664; *p* = 0.013). Half of the participants in the CEG group (*n* = 5, 50.0%) engaged in moderate physical activity and the remaining half of was engaged in vigorous exercise (*n* = 5, 50.0%), while 3 participants (27.3%) engaged in light physical activity and remaining 8 participants (72.7%) engaged in moderate physical activity in CG group. The distribution of post-surgery physical activity levels was significantly different between groups (Table 5).

**Table 5:** Post surgery changes in physical activity

Variable	Group	Pre-op		Post-op 4th month		Effect (*F*; *p*; ${{\varvec{\eta}}}_{\mathbf{p}}^{2}$)^a^		
		Mean ± SD	Median (IQR)	Mean ± SD	Median (IQR)	Group	Time	*G* × *T*
IPAQ score	CEG	543.50 ± 381.91	423.50 (347.00–693.00)	2676.70 ± 915.74	2746.00 (1956.00–3279.00)	***F* = 27.770; *p* < 0.001;** ${{\varvec{\eta}}}_{\mathbf{p}}^{2}$ **= 0.594**	***F* = 60.680; *p* < 0.001; ** ${{\varvec{\eta}}}_{\mathbf{p}}^{2}$** = 0.762**	***F* = 28.210; *p* < 0.001; ** ${{\varvec{\eta}}}_{\mathbf{p}}^{2}$** = 0.598**
	CG	491.82 ± 180.96	446.00 (330.00–660.00)	896.36 ± 459.42	876.00 (446.00–1272.00)			
**Pre-op IPAQ exercise level**		CEG (*n* = 10)		CG (*n* = 11)		Fisher’s exact test		
Low, *n* (%)		6 (60.0)		8 (72.7)		*p* = 0.659		
Moderate, *n* (%)		4 (40.0)		3 (27.3)				
**Post-op IPAQ exercise level**		CEG (*n* = 10)		CG (*n* = 11)		Pearson chi-square test		
Low, *n* (%)		0 (0.0)		3 (27.3)		**Chi-square = 8.664; *p*** = **0.013**		
Moderate, *n* (%)		5 (50.0)		8 (72.7)				
Vigorous, *n* (%)		5 (50.0)		0 (0.0)				

^a^Two-way ANOVA in repeated measures with aligned rank transformation. *G*, group; *T*, time; *CEG*, corrective exercise group; *CG*, control group; *SD*, standard deviation; *IQR*, interquartile range

### Changes in Nutrient Assessments

There were significant decreases in the energy intake, protein, fat, CHO, CHO percentage, fiber, B1, and iron intake of the participants 4 months post-surgery (*p* < 0.05). However, there were significant increases in protein percentage, vitamin D, and B12 intake post-surgery (*p* < 0.001) (Appendix Table 8). The increase in vitamin B1 intake was significantly lower in the CEG group than the CG group post-surgery (mean difference = 1.87; SE = 0.409; *t* = 3.876; Bonferroni *p* = 0.006; *d* = 1.324) (Table 6).

**Table 6:**
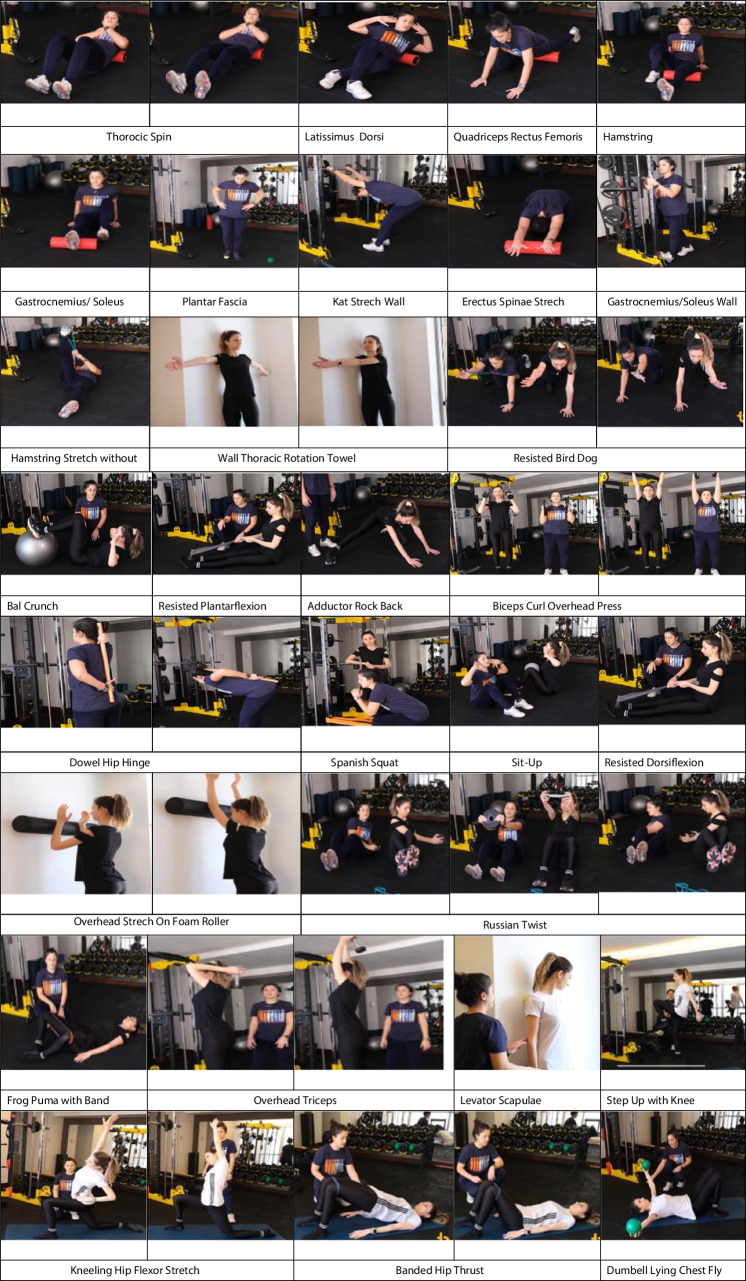
Visual Explanation of the Corrective Exercise Program Implemented in the Study

## Discussion

Participants in the CEG group showed positive improvements in the C.O.P.X. oscillation distance in open-eye static balance components and their levels of physical activity compared to CG group. It was also concluded that under the guidance of a dietitian, both groups developed healthier eating habits after bariatric surgery and reduced their consumption of sugary products, fatty foods, and packaged products. All participants had significant improvements in diet and body composition from before to 4 months post-surgery with no significant differences between corrective exercise and control groups. Studies indicate that physical activity undertaken after bariatric surgery supports long-term weight loss [3, 27, 28]. However, there are also studies suggesting an increase in lean body mass loss alongside fat mass loss [29].

In our study, when pre-op and post-op 4th-month measurements were compared, there were significant reductions in body weight (CEG: 25.59%; CG: 22.71%), fat mass (CEG: 42.38%; CG: 38.89%), fat percentage, internal fat rating, and waist circumference/height ratio in both groups, while the percentage of lean body mass significantly increased in both groups (CEG: 9.68%; CG: 6.13%).

The primary goal of corrective exercises is to enhance functional mobility, maintain body balance, improve coordination, and increase muscle strength to prevent potential movement disorders and imbalances following SG. In our study, a comprehensive corrective exercise program encompassing myofascial release, static flexibility, neuromuscular flexibility, isolated strength, and integration phases [30] did not create significant differences in body composition components between the groups over the 12-week period. Despite not prescribing a monitored exercise program for the CG, the increased motivation and health awareness following bariatric surgery, along with the increase in physical activity levels (IPAQ), and similar dietary habits, can be considered factors contributing to similar changes in body composition between the two groups.

There was no significant time-dependent difference in daily energy intake between the two groups at postoperative 4 months (CEG: 661.25 ± 178.23 kcal, CG: 698.63 ± 162.99 kcal). Furthermore, there were no differences between the two groups in terms of macro-nutrient consumption (protein, carbohydrates, and fat) at 4 months postoperatively (CEG: 60.01, 38.25, 29.80 g; CG: 66.25, 40.98, 29.97 g). Our research findings are consistent with similar studies [31, 32], indicating a decrease in the consumption of high-fat and high-sugar foods in the early months after surgery and no significant change in the preference for high-protein foods. Moreover, it is believed that dietitian follow-up has been effective in reducing the consumption of fatty foods (cheese and butter), carbohydrates (bread), sugary products (soft drinks, wafers, chocolates, desserts), and processed foods (chips, hamburgers, pizzas, french fries, pita, lahmacun, doner kebab) in both groups after surgery.

The finding in our study that the difference in eyes-open C.O.P.X. sway distance values between preoperative and postoperative 4 months was higher in the CG group than in the CEG group, along with a decreasing BMI, indicates an improvement in static balance parameters in the CEG group. Preoperatively, individuals with obesity have been shown to have higher COP displacement and sway speeds in the X-axis compared to non- individuals with obesity [9]. Handrigan et al. [33] found that there were no differences in visual and vestibular sensations among normal-weight, individuals with overweight or obesity, but variations in plantar mechanoreceptor sensitivity could be possible. This is because, with closed eyes, greater increases in postural sway speed were observed in individuals with obesity. Similarly, in our study, unlike GA measurements, a significant reduction in perimeter was observed postoperatively in the CG group. Kucuk Yetgin et al.’s [10] study revealed that while there was no significant difference in eyes-closed static balance during the rapid weight loss period before and after Laparoscopic Adjustable Gastric Banding Surgery at 6, 12, and 24 weeks, a statistically significant improvement in eyes-open static balance occurred at 6 weeks postoperatively.

The stringent selection of participants, characterized by uniform post-surgical profiles, while valuable for internal validity, may impede the extrapolation of our findings to a broader population. The study group assignment was based on participant’s willingness to exercise, therefore the post-surgery improvements in CEG could be affected by the high motivation of the participants who were willing to engage in the corrective exercise program. The necessity for physical presence due to the use of non-portable balance devices and the requirement for participants to be within proximity to the research center further constrained our sample diversity. Despite the successful use of online platforms to monitor and motivate adherence to the exercise protocol, the transient nature of motivation observed suggests that future research might benefit from exploring the comparative efficacy of remote versus in-person intervention strategies. Furthermore, the short duration of the study during a period of rapid weight loss limits our capacity to capture the long-term effects of the exercise regimen, and self-reported measures of physical activity and dietary intake must be interpreted with caution, given their inherent susceptibility to bias.

## Conclusion

In conclusion, our findings indicate that early home-based corrective exercises have a positive impact on the development of open-eye static balance, an important component of static balance in adult women with a BMI ≥ 30 kg/m^2^ after bariatric surgery. Although all participants, whether they performed corrective exercises or not, showed significant improvements in dietary preferences and body composition components. This improvement did not create a difference between the groups. Future studies are needed to investigate the effects of longer-term and/or different exercise programs.

## Appendix 1

Table 7

**Table 7:** Patient characteristics

Parameter	CEG (*n* = 10)	CG (*n* = 11)	p; test statistics; ES
Age, (year)^a^	40.80 ± 10.52	37.64 ± 11.00	*t* = − 0.672; *p* = 0.51^d^; *d* = − 0.294
Education level^b^			
Primary school^b^	1 (10.0)	0 (0.0)	${\chi }^{2}$= 2.836; *p* = 0.418^e^
High school^b^	1 (10.0)	4 (36.4)	
University^b^	7 (70.0)	6 (54.5)	
Postgraduate^b^	1 (10.0)	1 (9.1)	
Smoking status^b^			
Every day^b^	2 (20.0)	1 (9.1)	${\chi }^{2}$= 0.564; *p* = 0.754^e^
Not consumed for past 6 months^b^	2 (20.0)	3 (27.3)	
Non-smoker^b^	6 (60.0)	7 (63.6)	
TC (mg/dL)	1.20 ± 0.42^a^ 1.00 (1.00–1.00)^c^	1.27 ± 0.47^a^ 1.00 (1.00–1.50)^c^	W = 59.000; *p* = 0.739^f^; ${{\text{r}}}_{{\text{rb}}}$ = 0.073
HDL (mg/dL)	2.00 ± 0.00^a^ 2.00 (2.00–2.00)^c^	1.64 ± 0.51^a^ 2.00 (1.00–2.00)^c^	**W** = **35.000; *****p*** = **0.044;** ${\mathbf{r}}_{\mathbf{r}\mathbf{b}}$ = **0.364**
LDL (mg/dL)	1.10 ± 0.32^a^ 1.00 (1.00–1.00)^c^	1.36 ± 0.51^a^ 1.00 (1.00–2.00)^c^	W = 69.500; *p* = 0.182^f^; ${{\text{r}}}_{{\text{rb}}}$= 0.264
FPG (mg/dL)	1.30 ± 0.48 1.00 (1.00–1.75)^c^	1.18 ± 0.41^a^ 1.00 (1.00–1.00)^c^	W = 48.500; *p* = 0.567^f^; ${{\text{r}}}_{{\text{rb}}}$ = -0.118
Menopause presence^b^	2 (20.0)	1 (9.1)	${\chi }^{2}$= 0.509; *p* = 0.476^e^
MI story presence^b^	3 (30.0)	6 (54.5)	${\chi }^{2}$= 1.289; *p* = 0.256^e^
Hypertension presence^b^	1 (10.0)	3 (27.3)	${\chi }^{2}$= 1.014; *p* = 0.314^e^
Drug use^b^	5 (50.0)	3 (27.3)	${\chi }^{2}$= 1.147; *p* = 0.283^e^
Cortisone use^b^	4 (40.0)	3 (27.3)	${\chi }^{2}$= 0.382; *p* = 0.537^e^
Hypertension drug use^b^	1 (10.0)	2 (18.2)	${\chi }^{2}$= 0.286; *p* = 0.593^e^

Descriptive statistics presented with ^a^mean ± SD, ^b^frequency (percent), ^c^median(IQR). *CEG*, corrective exercise group; *CG*, control group; *ES*, effect size; *FPG*, fasting plasma glucose; *HDL*, high-density lipoprotein; *IQR*, interquartile range; *LDL*, low-density lipoprotein; *SD*, standard deviation; *TC*, total cholesterol; *d*, Cohen’s *d*; ${r}_{rb}$, rank-biserial correlation coefficient. Two-tailed *p*-value for ^d^Independent samples *T*-test; ^e^Pearson Chi-square test; ^f^Mann-Whitney *U* test

## Appendix 2

Table 8

**Table 8:** Post surgery changes in nutrients

Nutrient	G	Pre-op		Post-op 4th month		Effect (*F*;* p*; ${\eta }_{{\text{p}}}^{2})$ ^a^		
		Mean ± SD	Median(IQR)	Mean ± SD	Median(IQR)	Group	Time	*G* × *T*
Water (ml)	CEG	1875.00 ± 756.91	1750 (1250–2500)	1850.00 ± 851.47	1500 (1000–3000)	*F* = 0.001; *p* = 0.977; ${\eta }_{{\text{p}}}^{2}$ = 0.001	*F* = 0.651; *p* = 0.430; ${\eta }_{{\text{p}}}^{2}$ = 0.033	*F* = 0.436; *p* = 0.517; ${\eta }_{{\text{p}}}^{2}$ = 0.022
	CG	1977.27 ± 1063.34	2000 (1000–2500)	1727.27 ± 825.03	1500 (1000–2500)			
Energy (kcal)	CEG	3226.48 ± 454.97	3181.85 (2865.37–3685.6)	661.25 ± 178.23	659.68 (497.89–798.95)	*F* = 0.002; *p* = 0.965; ${\eta }_{{\text{p}}}^{2}$ = 0.001	***F*** = **396.674; *****p***** < 0.001;** ${{\varvec{\eta}}}_{\mathbf{p}}^{2}$ = **0.954**	*F* = 0.063; *p* = 0.804; ${\eta }_{{\text{p}}}^{2}$ = 0.003
	CG	3199.81 ± 606.68	3403.31 (2421.5–3633.66)	698.63 ± 162.99	643.16 (559.67–879.02)			
Protein (g)	CEG	123.25 ± 28.74	109.78 (105.09–145.77)	60.01 ± 25.16	56.08 (32.46–83.5)	*F* = 0.156; *p* = 0.697; ${\eta }_{{\text{p}}}^{2}$ = 0.008	***F*** = **35.790; *****p***** < 0.001;** ${{\varvec{\eta}}}_{\mathbf{p}}^{2}$ = **0.653**	*F* = 0.960; *p* = 0.339; ${\upeta }_{{\text{p}}}^{2}$ = 0.048
	CG	111.69 ± 29.21	112.9 (83.11–124.91)	66.25 ± 19.38	60.3 (50.24–92.3)			
Protein (%)	CEG	15.33 ± 2.98	14.83 (13.19–16.53)	35.11 ± 7.63	37.2 (27.46–41.8)	*F* = 0.229; *p* = 0.638; ${\eta }_{{\text{p}}}^{2}$ = 0.012	***F*** = **190.216; *****p***** < 0.001;** ${{\varvec{\eta}}}_{\mathbf{p}}^{2}$ = **0.909**	*F* = 1.387; *p* = 0.253; ${\eta }_{{\text{p}}}^{2}$ = 0.068
	CG	14.08 ± 3.20	13.54 (11.78–14.19)	37.55 ± 3.00	37.72 (36–39.4)			
Fat (g)	CEG	153.49 ± 39.92	146.76 (119.28–175)	29.80 ± 7.12	27.66 (26.51–34.21)	*F* = 0.318; *p* = 0.579; ${\eta }_{{\text{p}}}^{2}$ = 0.016	***F*** = **234.613; *****p***** < 0.001;** ${{\varvec{\eta}}}_{\mathbf{p}}^{2}$ = **0.925**	*F* = 0.368; *p* = 0.551; ${\eta }_{{\text{p}}}^{2}$ = 0.019
	CG	144.23 ± 30.45	127.97 (117–167.74)	29.97 ± 9.35	29.36 (23.17–41.37)			
Fat (%)	CEG	42.71 ± 8.09	40.96 (38.75–47.65)	42.14 ± 11.14	41.86 (32.39–43.91)	*F* = 1.266; *p* = 0.274; ${\eta }_{{\text{p}}}^{2}$ = 0.062	*F* = 0.380; *p* = 0.545; ${\eta }_{{\text{p}}}^{2}$ = 0.020	*F* = 0.164; *p* = 0.690; ${\eta }_{{\text{p}}}^{2}$ = 0.009
	CG	41.06 ± 7.10	40.35 (33.76–47.39)	38.30 ± 6.53	39.99 (34.06–42.11)			
CHO (g)	CEG	338.01 ± 68.44	316.54 (277.61–363.6)	38.25 ± 14.98	37.3 (28.76–52.08)	*F* = 0.524; *p* = 0.478; ${\eta }_{{\text{p}}}^{2}$ = 0.027	***F*** = **242.804; *****p***** < 0.001;** ${{\varvec{\eta}}}_{\mathbf{p}}^{2}$ = **0.572**	*F* = 0.332; *p* = 0.572; ${\eta }_{{\text{p}}}^{2}$ = 0.017
	CG	363.74 ± 105.45	405.79 (247.07–417.34)	40.98 ± 9.91	40.61 (32.69–50.25)			
CHO (%)	CEG	41.96 ± 5.93	43.94 (37.65–46.09)	22.75 ± 5.56	22.64 (19.43–27.84)	*F* = 1.079; *p* = 0.312; ${\eta }_{{\text{p}}}^{2}$ = 0.054	***F*** = **101.992; *****p***** < 0.001;** ${{\varvec{\eta}}}_{\mathbf{p}}^{2}$ = **0.843**	*F* = 0.145; *p* = 0.707; ${\eta }_{{\text{p}}}^{2}$ = 0.008
	CG	44.86 ± 7.63	45.94 (39.55–52.05)	24.14 ± 6.72	21.33 (20.17–28.55)			
Fiber (g)	CEG	30.42 ± 15.65	26.88 (21.37–34.06)	8.53 ± 4.75	9.34 (3.4–12.27)	*F* = 0.498; *p* = 0.489; ${\eta }_{{\text{p}}}^{2}$ = 0.026	***F*** = **65.277; *****p***** < 0.001;** ${{\varvec{\eta}}}_{\mathbf{p}}^{2}$ = **0.775**	*F* = 0.089; *p* = 0.768; ${\eta }_{{\text{p}}}^{2}$ = 0.005
	CG	33.53 ± 10.67	35.19 (30.65–41.18)	9.96 ± 2.82	9.07 (7.53–12.82)			
Vitamin D (mcg)	CEG	3.64 ± 2.03	2.48 (2.2–5.26)	18.77 ± 12.65	18.35 (4.91–31.6)	*F* = 0.707; *p* = 0.411; ${\eta }_{{\text{p}}}^{2}$ = 0.036	***F*** = **54.830; *****p***** < 0.001;** ${{\varvec{\eta}}}_{\mathbf{p}}^{2}$ = **0.743**	*F* = 0.144; *p* = 0.709; ${\eta }_{{\text{p}}}^{2}$ = 0.007
	CG	4.76 ± 2.31	4.62 (3.52–5.42)	21.53 ± 6.71	18.17 (16.64–31.61)			
Vitamin B1 (mg)	CEG	3.24 ± 1.60	3.35 (1.54–4.58)	1.65 ± 1.08	1.58 (0.32–2.73)	*F* = 0.806; *p* = 0.381; ${\eta }_{{\text{p}}}^{2}$ = 0.041	***F*** = **11.886; *****p*** = **0.003;** ${{\varvec{\eta}}}_{\mathbf{p}}^{2}$ = **0.385**	***F*** = **4.675; *****p*** = **0.044;** ${{\varvec{\eta}}}_{\mathbf{p}}^{2}$ = **0.197**
	CG	2.23 ± 1.33	1.9 (1.44–2.12)	1.87 ± 0.59	1.57 (1.48–2.73)			
Folate (mcg)	CEG	533.56 ± 219.72	492.73 (358.35–596.8)	553.80 ± 362.09	517.95 (130–917.4)	*F* = 0.073; *p* = 0.790; ${\eta }_{{\text{p}}}^{2}$ = 0.004	*F* = 1.529; *p* = 0.231; ${\eta }_{{\text{p}}}^{2}$ = 0.074	*F* = 0.872; *p* = 0.362; ${\eta }_{{\text{p}}}^{2}$ = 0.044
	CG	492.26 ± 109.04	497.8 (394–572.05)	637.55 ± 197.06	542.06 (513.75–917.2)			
Vitamin B12 (mcg)	CEG	6.93 ± 2.59	6.39 (5.5–8.88)	14.84 ± 8.25	12.76 (8.47–22.92)	*F* = 0.078; *p* = 0.784; ${\eta }_{{\text{p}}}^{2}$ = 0.004	***F*** = **22.842; *****p***** < 0.001;** ${{\varvec{\eta}}}_{\mathbf{p}}^{2}$ = **0.546**	*F* = 0.055; *p* = 0.817; ${\eta }_{{\text{p}}}^{2}$ = 0.003
	CG	6.96 ± 3.46	5.47 (4.65–9.08)	15.68 ± 5.38	13.27 (11.94–23.25)			
Iron (mg)	CEG	17.10 ± 5.25	15.18 (13.42–22.87)	12.24 ± 7.82	11.65 (3.11–20.02)	*F* = 0.169; *p* = 0.685; ${\eta }_{{\text{p}}}^{2}$ = 0.009	***F*** = **6.299; *****p*** = **0.021;** ${{\varvec{\eta}}}_{\mathbf{p}}^{2}$ = **0.249**	*F* = 0.483; *p* = 0.495; ${\eta }_{{\text{p}}}^{2}$ = 0.025
	CG	16.77 ± 2.95	17.34 (13.98–19.06)	14.02 ± 4.43	11.73 (11.12–20.52)			

^a^Two-way ANOVA in repeated measures with aligned rank transformation. *G*, group; *T*, time; *CEG*,corrective exercise group; *CG*, control group; *SD*, standard deviation; *IQR*, interquartile range
